# African Swine Fever Virus MGF 360-2L Disrupts Host Antiviral Immunity Based on Transcriptomic Analysis

**DOI:** 10.3390/vaccines13090918

**Published:** 2025-08-28

**Authors:** Taoqing Zhang, Xiaodong Qin, Sujie Dong, Yuanshu Wu, Xiaolan Qi, Jingjing Ren, Yuan Wen, Zhengwang Shi, Tao Feng, Bingjie Sun, Changying Wang, Haixue Zheng

**Affiliations:** 1 State Key Laboratory for Animal Disease Control and Prevention, College of Veterinary Medicine, Lanzhou University, Lanzhou Veterinary Research Institute, Chinese Academy of Agricultural Sciences, Lanzhou 730046, China; 2 Gansu Province Research Center for Basic Disciplines of Pathogen Biology, Lanzhou 730046, China

**Keywords:** African swine fever virus, *MGF 360-2L*, RNA-Seq, recombinant virus functional analysis, host antiviral immunity

## Abstract

**Background/Objectives:** The African swine fever virus (ASFV) multi-gene family (MGF) 360 proteins play critical roles in immune evasion, replication regulation, and virulence determination. Despite substantial advances in this field, the functional roles of many members within this gene family remain to be fully characterized. **Methods:** In this study, Transcriptional kinetics analysis indicated that the expression profile of *MGF 360-2L* was consistent with that of the late marker gene *B646L* (p72). Transcriptomic profiling identified 13 and 171 differentially expressed genes (DEGs) at 12 and 24 h post-infection (hpi) with Δ*MGF 360-2L*, respectively. **Results:** Gene Ontology (GO) and Kyoto Encyclopedia of Genes and Genomes (KEGG) pathway enrichment analyses indicated that these DEGs were predominantly enriched in Type I interferon (IFN-I) signaling pathways. It is noteworthy that transcriptome analysis further demonstrates that the absence of *MGF 360-2L* specifically results in the dysregulation of expression of the replication-essential genes *E199L* and *E301R*. These findings indicate that *MG F360-2L* is essential for maintaining the stable expression of these proteins. **Conclusions:** *MGF 360-2L* is a late gene that contributes to the precise regulation of viral protein expression and modulates the host immune response during infection.

## 1. Introduction

African swine fever (ASF) is a highly contagious and fatal viral disease of swine caused by the African swine fever virus (ASFV) [1]. It affects domestic pigs and wild boars across all breeds and age groups, and is characterized by high morbidity and mortality rates [2,3]. At present, no commercially available vaccines or clinically effective anti-ASFV therapeutics exist for the prevention or treatment of this disease [4]. Consequently, ASF remains endemic in many regions worldwide, posing a significant threat to global food security and the sustainability of the livestock industry [5].

ASFV is a member of the Asfarviridae family and belongs to the group of nucleo-cytoplasmic large DNA viruses (NCLDVs), representing the only known DNA arbovirus [6]. It is a large, enveloped double-stranded DNA (dsDNA) virus with a linear dsDNA genome ranging in size from approximately 170 to 193 kbp, capable of encoding about 150 to 200 distinct proteins [7,8]. The ASFV genome contains multiple unique and functionally significant multigene families (MGF100, MGF110, MGF300, MGF360, and MGF530/505) [9,10]. Among these, the 360 family proteins of ASFV, also referred to as multigene family 360 (MGF360), are localized in the highly variable regions at both the left and right termini of the ASFV genome [11]. These proteins constitute a group of highly divergent non-structural proteins that play critical roles in viral immune evasion, host range modulation, and virulence determination. Different members of the MGF360 family contribute to viral survival and replication by targeting multiple critical components of the host immune system. Specifically, *MGF 360-16R*, a mitochondrial-targeting protein, potently induces mitochondrial-dependent apoptosis [12]. *MGF 360-12L* functions as a multifunctional immune evasion protein capable of simultaneously inhibiting type I interferon (IFN-I), NF-κB, and JAK/STAT signaling pathways, primarily through disruption of the interaction between importin α and NF-κB, thereby suppressing IFN-I production [13]. *MGF 360-4L* inhibits the phosphorylation of IRF3, preventing its nuclear translocation and consequently attenuating the IFN-β response [14]. *MGF 360-9L*, recognized as a key virulence factor, suppresses the IFN-β signaling pathway by promoting the degradation of STAT1 and STAT2 [15]. Moreover, *MGF 360-11L* modulates IFN-I expression through negative regulation of the cGAS signaling pathway [16]. Collectively, these findings demonstrate that the MGF360 family proteins employ a diverse array of mechanisms—including suppression of IFN-I production, blockade of JAK/STAT signaling, interference with the cGAS-STING pathway, and induction of apoptosis—to collectively facilitate ASFV immune evasion.

Here, we report that the transcription of *MGF 360-2L* predominantly occurs during the late phase of ASFV infection. Transcriptome analysis reveals that the presence of *MGF 360-2L* is associated with enhanced interferon-related transcriptional signatures. Compared to the ASFV CN/GS/2018, the ASFV CN/GS/2018-ΔMGF360-2L significantly downregulated the expression of multiple immune-related genes during infection of Porcine alveolar macrophages (PAMs). Between 12 hpi and 24 hpi, the most significantly suppressed genes included several chemokines (e.g., *CXCL10* and *CXCL11*), type I interferons (e.g., *IFNA1* and *IFNB1*), and *SOCS1*, a key negative regulator of the JAK-STAT signaling pathway [17], *SOCS1* overexpression results in the suppression of T cell and macrophage functions. Furthermore, *MGF 360-2L* demonstrates a strong association with two essential viral genes, *E199L* [18] and *E301R* [19]. These findings contribute to a deeper understanding of the immune evasion strategies employed by ASFV, indicating that the virus may modulate the host immune response through a complex regulatory network. Further investigations are warranted to elucidate the precise molecular mechanisms by which *MGF 360-2L* regulates IFN-I signaling and to determine its specific role in the pathogenesis of ASFV.

## 2. Materials and Methods

### 2.1. Viruses and Cells

PAMs were isolated using established bronchoalveolar lavage techniques and maintained in RPMI 1640 medium (Gibco, New York, NY, USA), supplemented with 10% heat-inactivated porcine serum (Sigma-Aldrich, Darmstadt, Germany) and 1% penicillin-streptomycin-gentamicin antibiotic cocktail (Solarbio, Beijing, China), under standard cell culture conditions of 37 °C, 5% CO_2_, and saturated humidity within a Thermo Scientific cell incubator. Wild-type African swine fever virus (ASFV CN/GS/2018-WT) and the deleted ASFV(ASFV CN/GS/2018-ΔMGF 360-2L) were propagated in PAMs according to the previously published protocol [20]. The resulting viral suspension was aliquoted and stored at −80 °C for use in subsequent experimental procedures.

### 2.2. Generation and Identification of ASFV CN/GS/2018-ΔMGF-360-2L

To generate the *ASFV CN/GS/2018-ΔMGF 360-2L* gene deletion mutant strain, a transfer vector was designed and constructed based on the principle of homologous recombination. This vector comprises the following key components: (1) the left and right homologous arms corresponding to the *MGF 360-2L* gene locus in the ASFV CN/GS/2018 genome; and (2) an EGFP reporter gene expression cassette under the control of the p72 promoter. As a result of this design, the *MGF 360-2L* gene will be replaced by the p72-EGFP expression cassette. The detailed construction procedure is as follows: First, using the cDNA of ASFV CN/GS/2018 as a template, the left and right homologous arm fragments of the *MGF 360-2L* gene were amplified via PCR with specific primers containing designated restriction enzyme sites (as listed in Table 1). Subsequently, overlapping PCR was employed to fuse these fragments with the p72-EGFP expression cassette. The resulting fusion fragment was then cloned into the pUC57 vector. Following sequence confirmation, the recombinant plasmid was designated as pUC57-p72-ΔMGF 360-2L-EGFP. The recombinant virus generated through homologous recombination was purified by serial limiting dilution on PAM monolayers. The purified ASFV CN/GS/2018-ΔMGF 360-2L strain was subsequently expanded in PAMs to generate a viral stock. To confirm the absence of residual wild-type sequences in each recombinant viral genome, viral DNA was extracted from ASFV CN/GS/2018-ΔMGF 360-2L infected PAMs and subjected to PCR analysis using gene-specific primers (as listed in Table 1).

### 2.3. Characteristic of the MGF 360-2L Gene Expression

PAMs were prepared in 12-well plates and infected with ASFV CN/GS/2018 at a multiplicity of infection (MOI) of 1.0. PAMs subjected to mock infection were used as control samples. Samples were collected at 0, 3, 6, 9, 15, 18, 21 and 24 hpi, with three biological replicates per time point. Total RNA was extracted from the PAMs using NucleoZOL (MACHEREY-NAGEL, Düren, Germany) reagent and reverse-transcribed into complementary DNA using the 5X HiScript II qRT Super Mix (Vazyme, Nanjing, China). Real-time fluorescence quantitative PCR based on SYBR Green I was performed to amplify specific fragments of the *B646L*, *CP204L*, and *MGF 360-2L* genes, as well as the internal reference gene *GAPDH*. Experimental data were analyzed using GraphPad Prism version 8.0.2. The sequences of the primers used are provided in Table 2.

### 2.4. RNA-Seq Analysis

Infections were performed within the designated time frame using ASFV CN/GS/2018 or ASFV CN/GS/2018-ΔMGF 360-2L, followed by total RNA extraction using the NucleoZOL reagent. RNA sample concentration and purity were assessed using a NanoDrop 2000 spectrophotometer (Thermo Fisher Scientific, Waltham, MA, USA), while RNA integrity was evaluated using an Agilent 2100 Bioanalyzer in conjunction with the 2100 RNA Nano 6000 Assay Kit (Agilent Technologies, Palo Alto, CA, USA). Following completion of the quality control (QC) procedure, poly-A-containing mRNA was enriched using the TIANSeq mRNA Capture Kit (TIANGEN, Beijing, China). The enriched RNA was subsequently used as a template for sequencing library construction using the TIANSeq Fast RNA Library Kit (Illumina, San Diego, CA, USA). Briefly, the transcriptome library preparation involved RNA fragmentation, first-strand and second-strand cDNA synthesis, end repair, A-tailing, adapter ligation, fragment size selection, and PCR amplification of the library. Transcriptomic data were analyzed using the Kolmogorov–Smirnov test implemented in the topGO R (Version is 2.42.0.)package to identify DEGs. After correction for multiple hypothesis testing, GO terms with adjusted *p* values less than 0.05 were considered significantly enriched and further analyzed for enrichment in KEGG pathways using the “clusterProfiler” software package (version 4.2.1).

### 2.5. Screening and Validation of Differentially Expressed Transcripts

To validate the reliability of RNA-Seq high-throughput sequencing results, selected up-regulated and down-regulated genes were subjected to RT-qPCR analysis. The primer sequences used are listed in Table 3. (RNA samples were processed using the One-Step QuantiNova SYBR PCR Mix Kit (QIAGEN, Düsseldorf, Germany) following the manufacturer’s instructions for one-step reverse transcription and quantitative PCR. Real-time fluorescence quantitative PCR was conducted on a LightCycler 480 II system (Roche, Basel, Switzerland). Each reaction contained 5 µg of RNA in a total volume of 20 µL. Thermal cycling conditions were as follows: initial denaturation at 95 °C for 2 min, followed by 40 cycles of denaturation at 95 °C for 5 s and annealing/extension at 60 °C for 10 s. All samples were analyzed in triplicate to ensure reproducibility. *GAPDH* was employed as the internal reference housekeeping gene.

### 2.6. Virus Titration

Following infection of PAMs with ASFV CN/GS/2018 and ASFV CN/GS/2018-ΔMGF 360-2L at predetermined time points, the cells along with their culture medium were subjected to repeated freeze–thaw cycles at −80 °C and subsequently diluted. The infected PAMs were then seeded into 96-well plates, and corresponding sample aliquots were added to each well for 10-fold serial dilution. The titration procedure was performed in triplicate. On day 7 post-inoculation, the tissue culture infectious dose 50% (TCID_50_) was assessed, and the 50% effective infectious dose (TCID_50_) was calculated using the method described by Reed and Muench. Virus growth kinetics were analyzed and plotted using GraphPad Prism software.

### 2.7. Facility Biosafety Statement

All live experiments involving ASFV were conducted in the enhanced biosafety level 3 (BSL-3) facility at the Lanzhou Veterinary Research Institute (LVRI), Chinese Academy of Agricultural Sciences. All experimental procedures were carried out in compliance with national regulations and had received prior approval from both the Ministry of Agriculture and Rural Affairs and the China National Accreditation Service for Conformity Assessment (CNAS).

### 2.8. Statistical Analysis

All graphs were generated using GraphPad Prism 8.0 (USA). Statistical comparisons between groups were performed with one-way analysis of variance (ANOVA) using Statistica 17.0 software (StatSoft, Street Tulsa, OK, USA). Differences were considered statistically significant when * *p* < 0.05, ** *p* < 0.01, or *** *p* < 0.001, and non-significant when *p* > 0.05.

## 3. Results

### 3.1. Construction of ASFV CN/GS/2018-ΔMGF 360-2L

**Figure 1:**
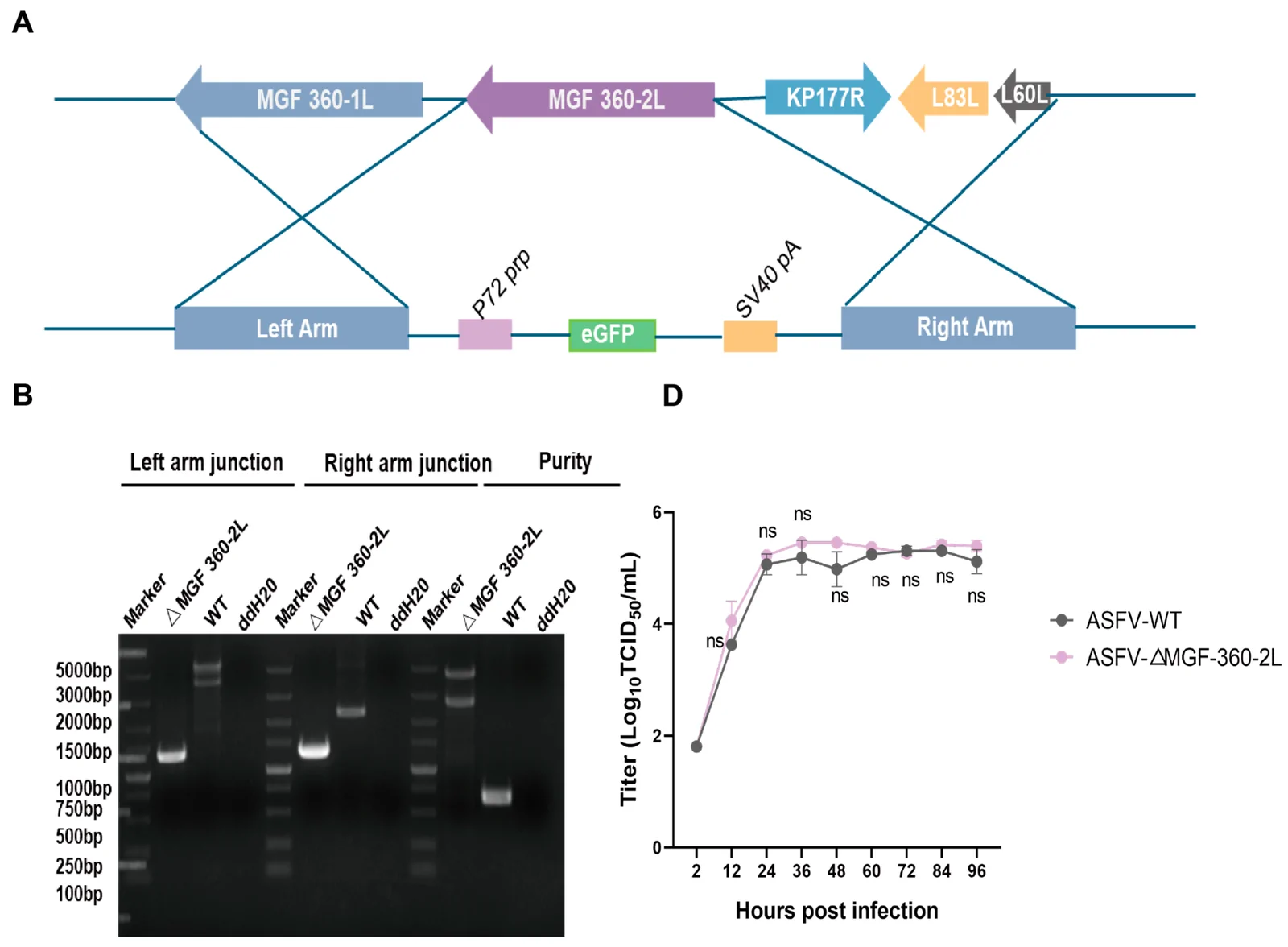

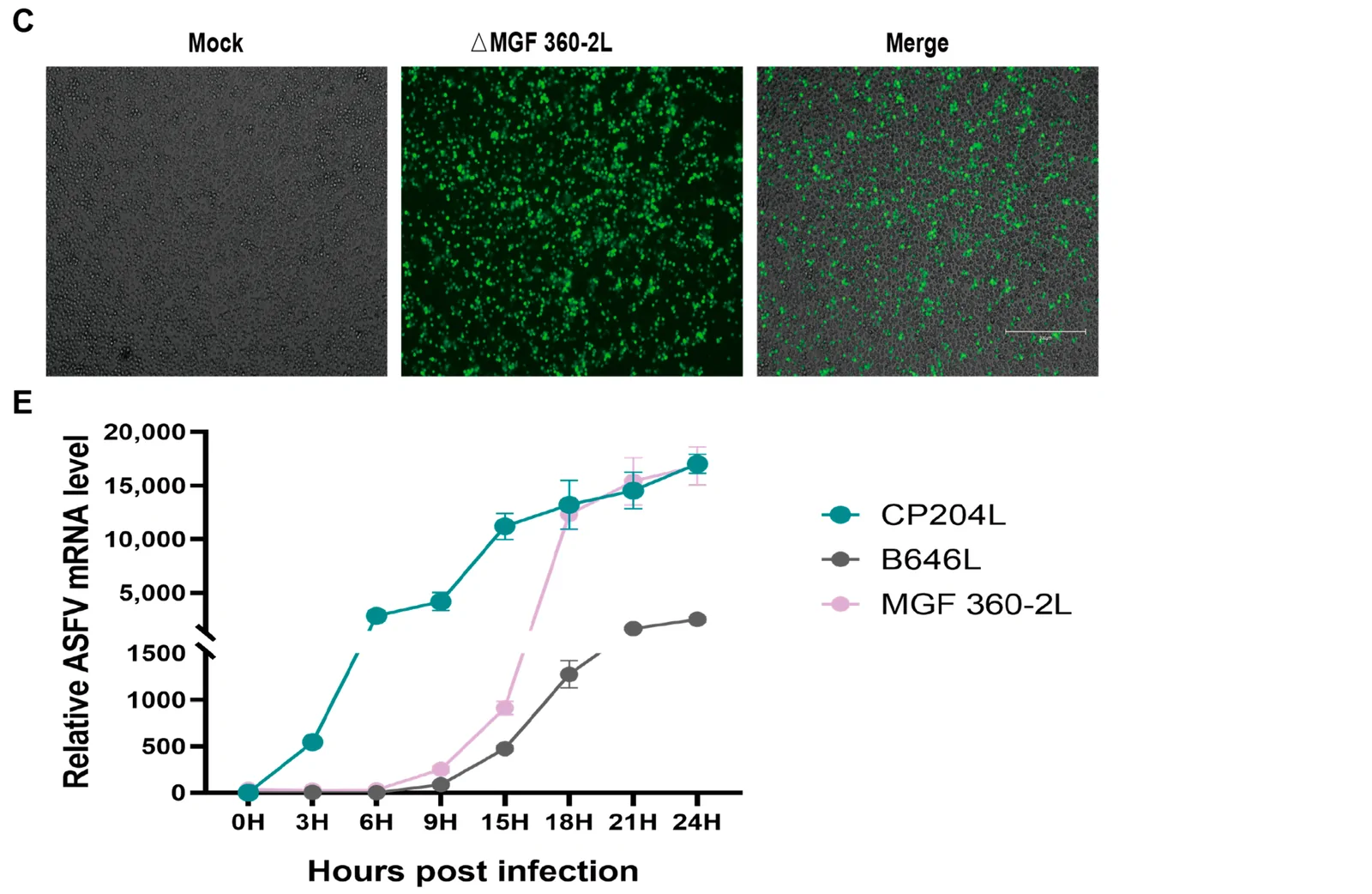
Construction of ASFV CN/GS/2018-ΔMGF 360-2L. (**A**) Schematic diagram of the construction of the *MGF 360-2L* deletion mutant. (**B**) The deletion of the ASFV *MGF 360-2L* gene was confirmed by PCR amplification. (**C**) PAMs were infected with purified ASFV CN/GS/2018-ΔMGF 360-2L,Scale bars,300μm. (**D**) Cells were infected with either ASFV-WT or ASFV-ΔMGF 360-2L (MOI = 1), and samples were collected at 2, 12, 24, 36, 48, 60, 72, 84, and 96 h post-infection (hpi). Viral titers were determined using the TCID50 assay, no significant difference, *p* > 0.05. (**E**) The relative expression levels of *MGF 360-2L*, *CP204L*, and *B646L* mRNA are displayed. Specific primers targeting *MGF 360-2L*, *CP204L*, *B646L*, and the internal reference gene *GAPDH* were employed to measure the average cycle threshold (Ct value) at 0, 3, 6, 9, 15, 18, 21 and 24 hpi following infection of PAMs with ASFV CN/GS/2018 (MOI = 1.0), using quantitative reverse transcription PCR (qRT-PCR).

Members of the MGF360 family play critical roles in viral immune evasion, host range regulation, and virulence determination by targeting multiple key components of the host immune system. The *MGF 360-2L* gene is located between nucleotide positions 2022 and 3110 in the viral genome and is flanked by the *MGF 360-1L* and *KP177R* genes, respectively (Figure S1A). The *MGF 360-2L* gene of ASFV genotype II encodes a 363 aminoacid peptide that lacks a transmembrane domain (Figure S1B), and its specific function remains to be elucidated. To further elucidate the functional role of the ASFV *MGF 360-2L* gene, we generated a recombinant virus strain with deletion of the *MGF 360-2L* gene. As shown in (Figure 1A), the construction strategy involved inserting an enhanced green fluorescent protein (EGFP) expression cassette under the control of the ASFV p72 promoter (p72-EGFP) into the viral genome. The recombinant virus, designated as ASFV CN/GS/2018-ΔMGF 360-2L, was propagated in PAMs. Fluorescence signals were monitored to confirm successful viral replication. Subsequently, the virus was purified through endpoint dilution, and the final purified viral population was expanded in PAMs culture system to generate the working virus stock. Subsequently, PCR amplification analysis was performed using newly designed specific primers targeting both purity assessment and insertion confirmation (Figure S2). Following 10 rounds of endpoint dilution, the PCR results demonstrated that the ASFV CN/GS/2018-ΔMGF 360-2L viral strain remained free from contamination by the wild-type ASFV CN/GS/2018 strain (Figure 1B). Meanwhile, fluorescence microscopy observations revealed that the ASFV-ΔMGF 360-2L mutant strain was marked with EGFP and could emit green fluorescence, thus enabling the tracking of the virus (Figure 1C). In addition, the growth characteristics of ASFV-ΔMGF 360-2L in PAMs were similar to those of ASFV-WT (Figure 1D). Finally, to investigate the transcriptional profile of *MGF 360-2L*, the ASFV CN/GS/2018 strain was employed to infect PAMs in this study. The expression dynamics of this gene were dynamically monitored using RT-PCR. Concurrently, the early viral marker gene *CP204L* and the late marker gene *B646L* were assessed as reference controls. The results demonstrate (Figure 1E) that *CP204L* mRNA accumulates rapidly within 1 to 3 h post-infection, consistent with the expression pattern of typical early genes. Notably, the transcriptional kinetics of *MGF 360-2L* and *B646L* exhibit a high degree of similarity, with both mRNA levels showing marked increases after 6 h post-infection. This transcriptional profile, which aligns with that of late marker genes, indicates that *MGF 360-2L* is a late-expressed gene of the ASFV CN/GS/2018 strain.

### 3.2. RNA-Seq Data Quality Analysis

PAMs were infected with ASFV CN/GS/2018 or ASFV CN/GS/2018-ΔMGF 360-2L at an MOI of 1.0 for 12 and 24 h, respectively. RNA sequencing was subsequently performed to assess the impact of the ASFV *MGF 360-2L* gene deletion on the expression profiles of associated genes. Uninfected PAMs served as the mock control group. Subsequently, total RNA was extracted from each treatment group and subjected to transcriptome sequencing to assess the impact of *MGF 360-2L* gene deletion on the host gene expression profile. RNA samples from the five experimental groups (mock control group, WT-ASFV 12 hpi, WT-ASFV 24 hpi, ΔMGF 360-2L 12 hpi, and ΔMGF 360-2L 24 hpi) were evaluated for purity, concentration, and integrity using Nanodrop, Qubit 2.0, and Agilent 2100 instruments.

The results indicated that both the concentrations and total quantities of the five RNA samples fulfilled the sequencing requirements. Sequencing was performed on the HiSeq 2500 high-throughput sequencing platform. Base composition and quality analysis revealed that the base distribution of the raw sequencing data was appropriate across all samples, with the proportion of Q30 base quality values being no lower than 94.92% (Table 4).

The sequencing data from each sample were aligned to the selected reference genome sequence. Statistical analysis of the alignment results revealed that the alignment efficiency of reads across all samples ranged from 86.94% to 94.64%, with the data utilization rate falling within the normal range (see Table 5). These findings indicate that the selected reference genome is appropriate for downstream analysis.

### 3.3. Host DEG Ontology Analysis

Compared with the ASFV CN/GS/2018 infection group, 13 genes were significantly differentially expressed at 12 hpi, with 8 genes down-regulated and 5 up-regulated. Similarly, at 24 hpi, 171 significantly differentially expressed genes were identified, comprising 136 down-regulated and 35 up-regulated genes (Log2FC > 1 or <−1 and padj < 0.05). Further, GO functional annotation and KEGG pathway analyses were performed on these differentially expressed genes.

**Figure 2:**
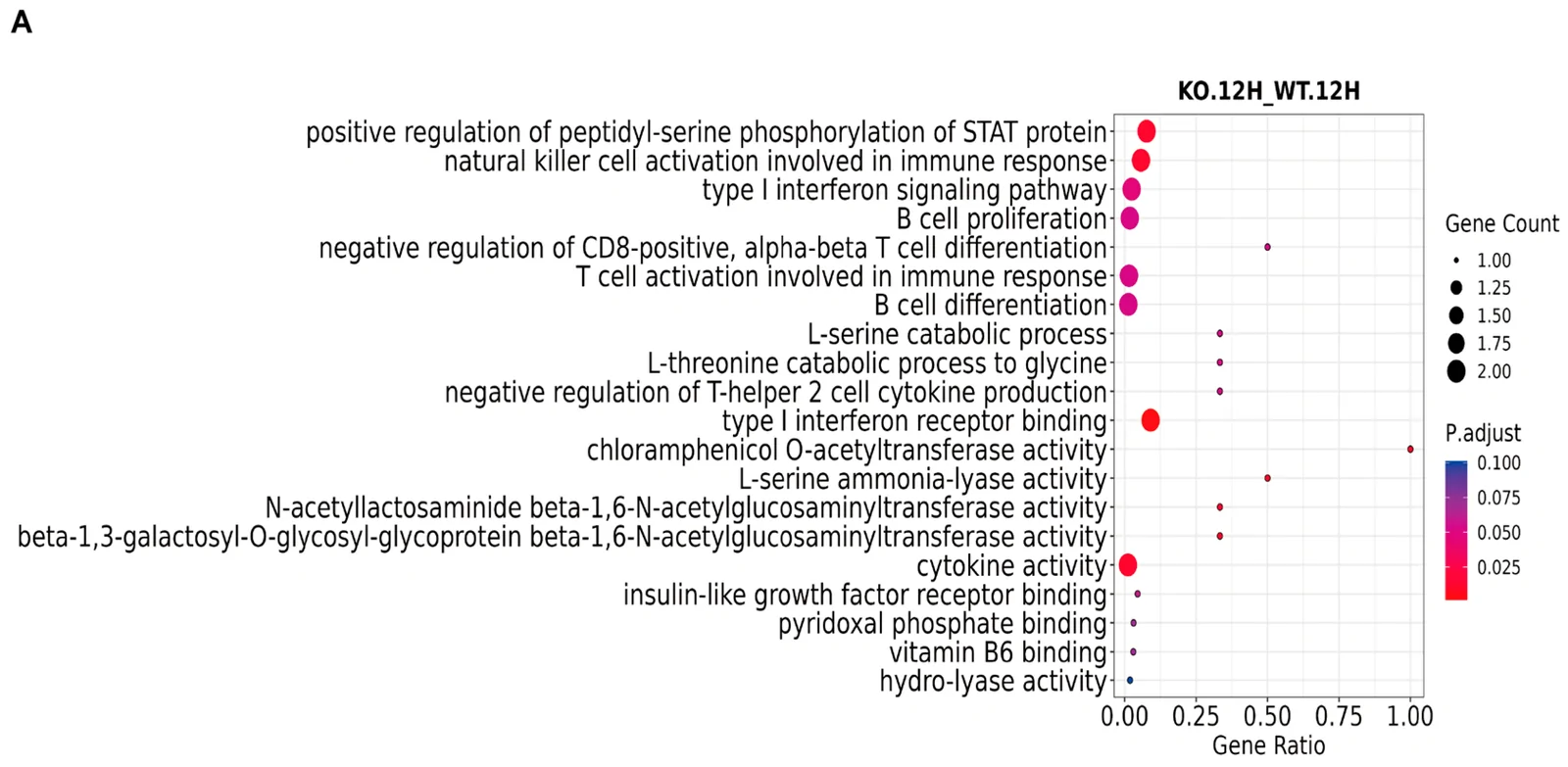

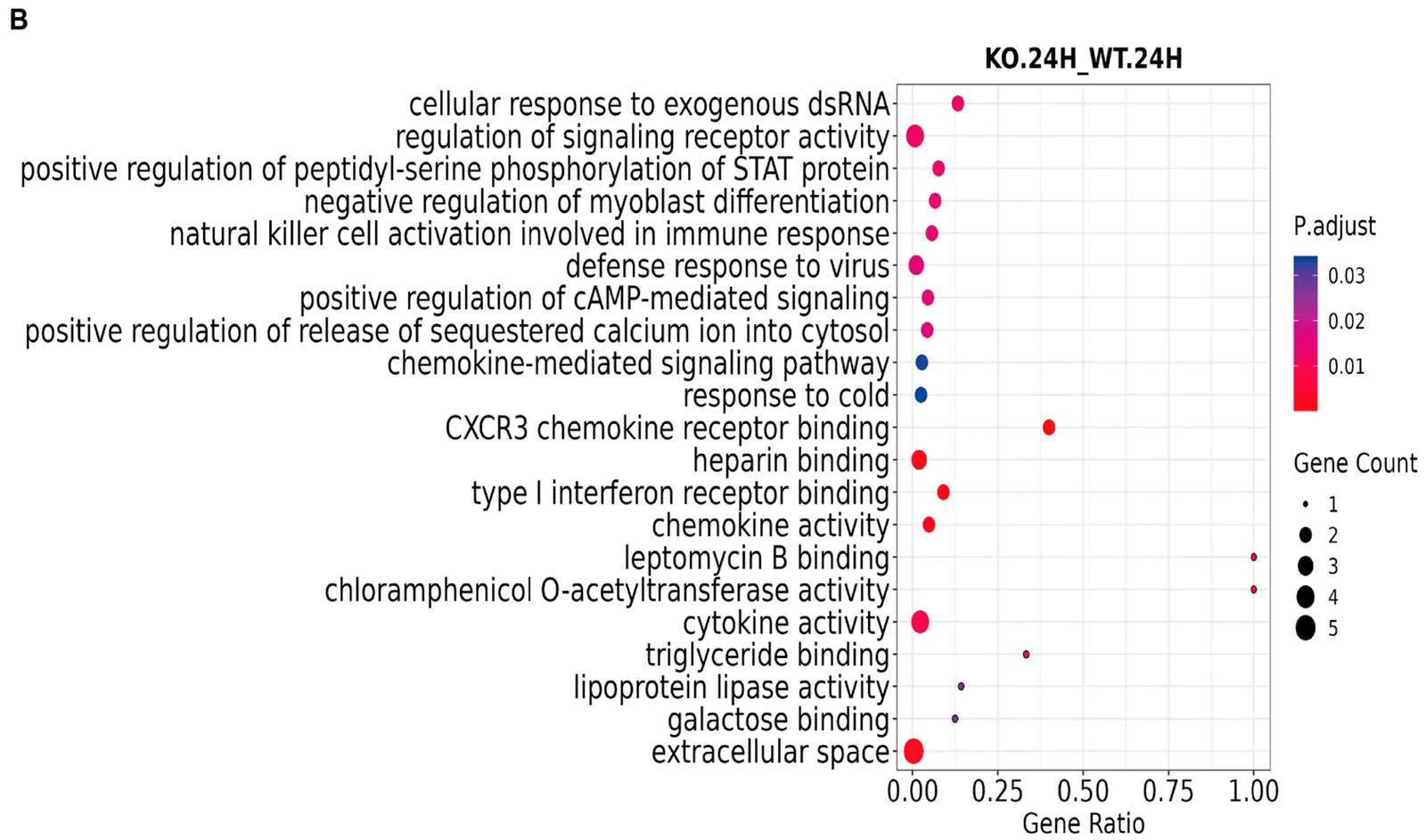
GO analysis of ASFV CN/GS/2018-ΔMGF 360-2L-infected PAMs through RNA-seq. (**A**) GO analysis of ASFV CN/GS/2018-ΔMGF 360-2L vs. ASFV CN/GS/2018 at 12 hpi. (**B**) GO analysis of ASFV CN/GS/2018-ΔMGF 360-2L vs. ASFV CN/GS/2018 at 24 hpi.

GO functional enrichment analysis systematically characterizes, classifies, and describes gene functions from multiple perspectives through the use of three major ontologies: biological process (BP), cellular component (CC), and molecular function (MF). The results demonstrate that, in the biological process category, significantly differentially expressed genes are predominantly associated with signaling pathways and defense responses to viral infection. With regard to cellular component, these genes primarily encode extracellular region proteins localized on the outer leaflet of the plasma membrane. In the molecular function category, they are mainly involved in chemokine activity and CCR chemokine receptor binding (Figure 2A,B).

### 3.4. Kyoto Encyclopedia Analysis of Host DEGs

**Figure 3:**
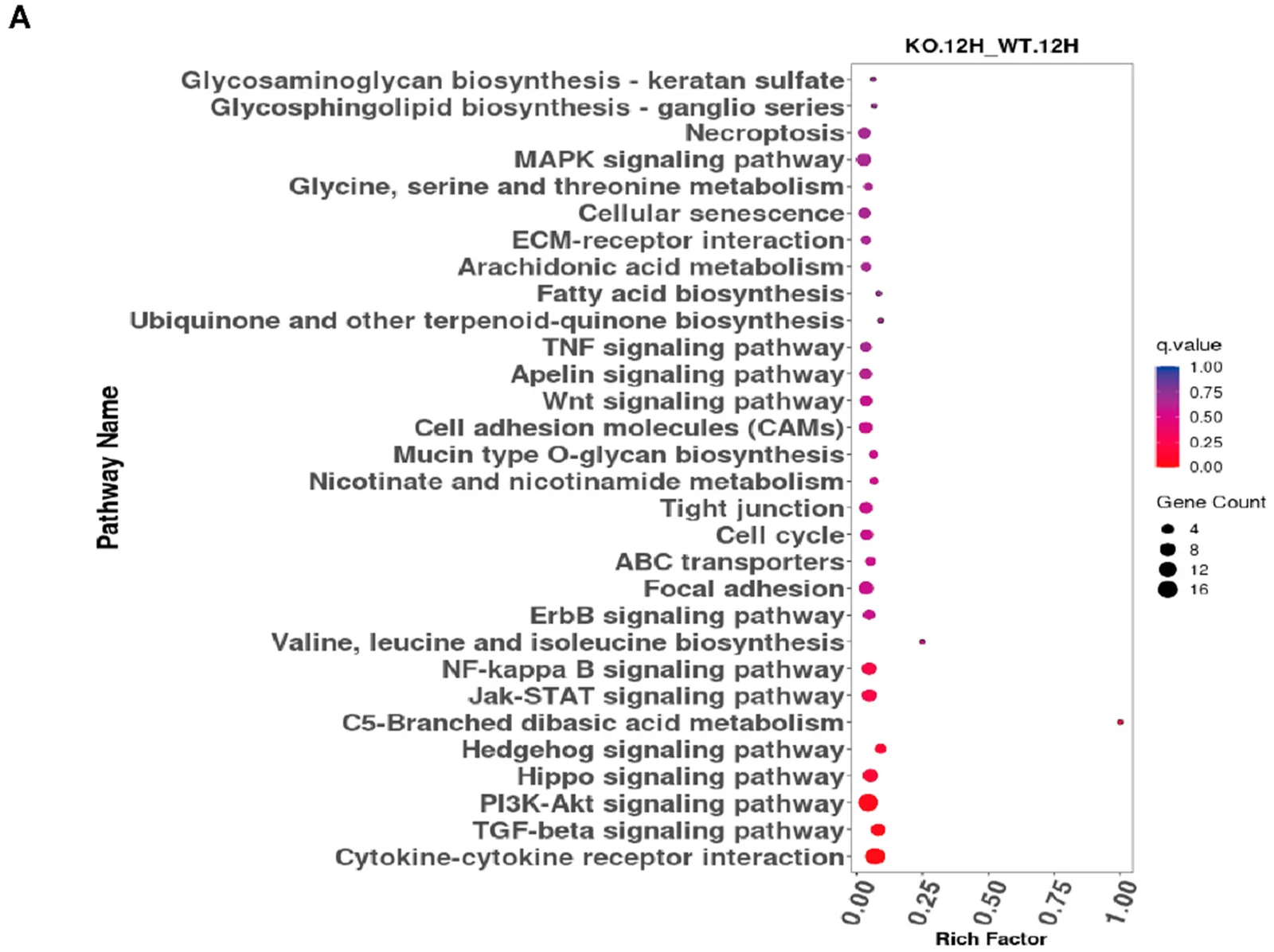

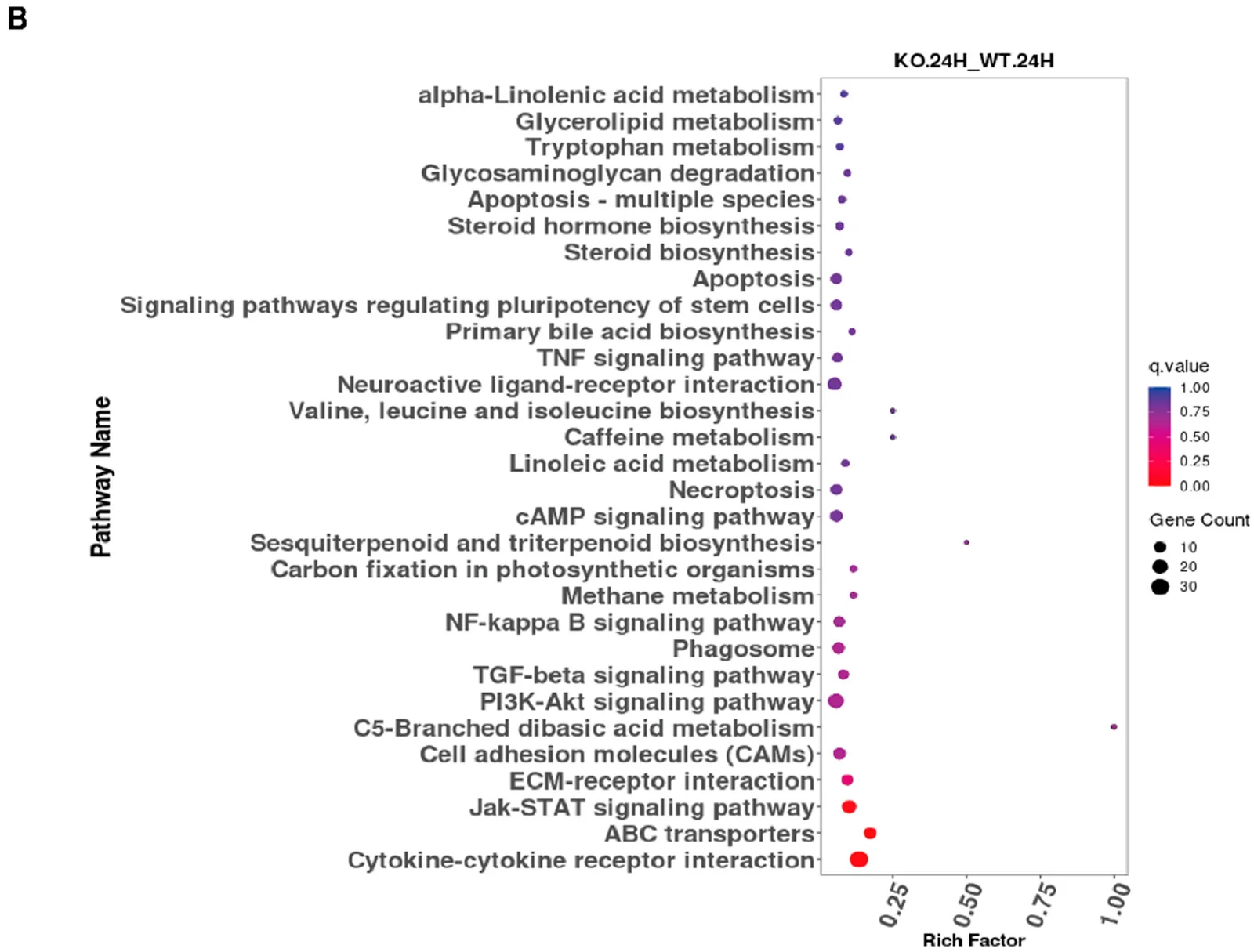
KEGG analysis of ASFV CN/GS/2018-ΔMGF 3 60-2L-infected PAMs through RNA-seq. (**A**) KEGG analysis of ASFV CN/GS/2018-ΔMGF 360-2L vs. ASFV CN/GS/2018 at 12 hpi. (**B**) KEGG analysis of ASFV CN/GS/2018-ΔMGF 360-2L vs. ASFV CN/GS/2018 at 24 hpi.

The KEGG database serves as a comprehensive resource platform that integrates genomic, chemical, and systems-level functional information, with the aim of enabling a more thorough understanding of the molecular reaction networks associated with encoded genes. The data indicate that significantly differentially expressed genes are predominantly enriched in the NF-κB signaling pathway, TGF-β signaling pathway, Jak-STAT signaling pathway, and cytokine-cytokine receptor (CCR) interaction pathway (Figure 3A,B).

### 3.5. Transcriptome Validation of Host Genes

**Figure 4:**
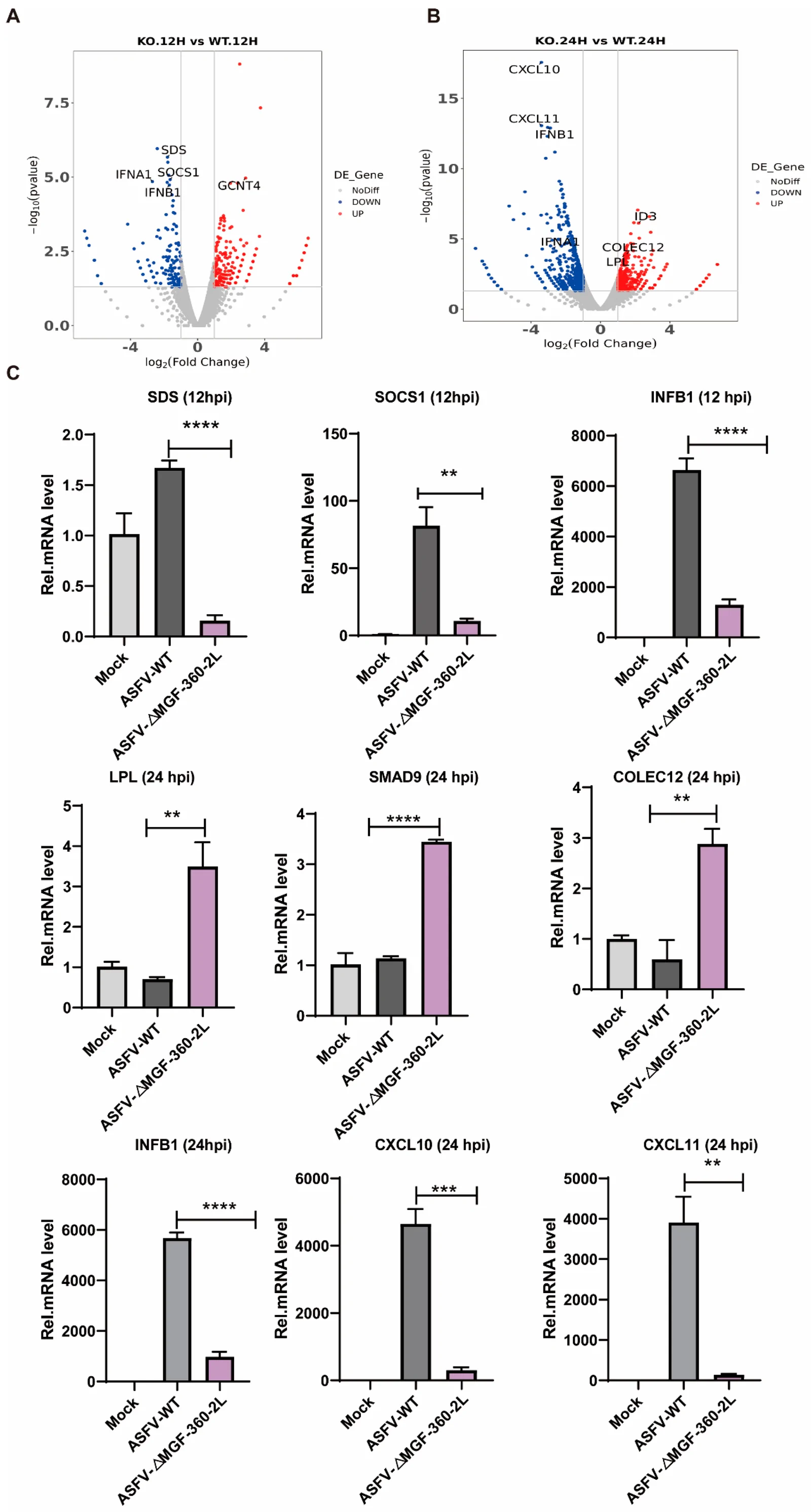
Validation of RNA-Seq results by RT-qPCR. (**A**) Volcano plot of ASFV CN/GS/2018-ΔMGF 360-2L vs. ASFV CN/GS/2018 at 12 hpi. (**B**) Volcano plot of ASFV CN/GS/2018-ΔMGF 360-2L vs. ASFV CN/GS/2018 at 24 hpi. (**C**) PAMs were uninfected or infected with ASFV CN/GS/2018 or ASFV CN/GS/2018-ΔMGF 360-2L at 1.0 MOI for 12 hpi or 24 hpi. RNA was extracted and RT-qPCR was carried to test their expression level. The data are presented as means with SD. *p*-values were analyzed by Student’s *t*-test.** *p* <  0.01, *** *p* <  0.001, **** *p* < 0.0001 and non-significant at *p*  >  0.05.

Compared with the control group (ASFV CN/GS/2018), infection with ASFV CN/GS/2018-ΔMGF 360-2L resulted in a significant down-regulation of immune-related genes in PAMs. At 12 hpi, *SDS* exhibited the most pronounced down-regulation, followed by *SOCS1*, a key negative regulator of cytokine signaling, with *IFNA1* and *IFNB1* ranked third and fourth, respectively (Figure 4A). By 24 hpi, the most significantly suppressed genes included the chemokines *CXCL10* and *CXCL11*, as well as *IFNA1* and *IFNB1* (Figure 4B). To validate these observations, RT-qPCR was conducted using RNA samples from PAMs infected with either wild-type ASFV or the *MGF 360-2L* deletion mutant at 12 and 24 hpi. The data demonstrate that gene expression undergoes dynamic alterations during the infection process: at 12 h post-infection, the expression of *SDS*, *SOCS1*, and *IFNB1* is significantly downregulated; by 24 h post-infection, *IFNB1*, *CXCL10*, and *CXCL11* exhibit further downregulation, whereas the expression levels of *LPL*, *SMAD9*, and *COLEC12* are upregulated. Specifically, *LPL* functions as a key enzyme in lipid metabolism and plays a central role in lipoprotein metabolism [21]; *SMAD9* serves as a core signal transduction protein within the TGF-β signaling pathway [22]; and *COLEC12*, as a pattern recognition molecule, contributes to immune responses through activation of the complement system [23]. These findings are highly consistent with the RNA-seq data, revealing a coordinated regulatory pattern of genes associated with lipid metabolism, signal transduction, and innate immunity during the later stages of infection (Figure 4C).

### 3.6. Transcriptome Analysis and Validation of ASFV Genes

**Figure 5:**
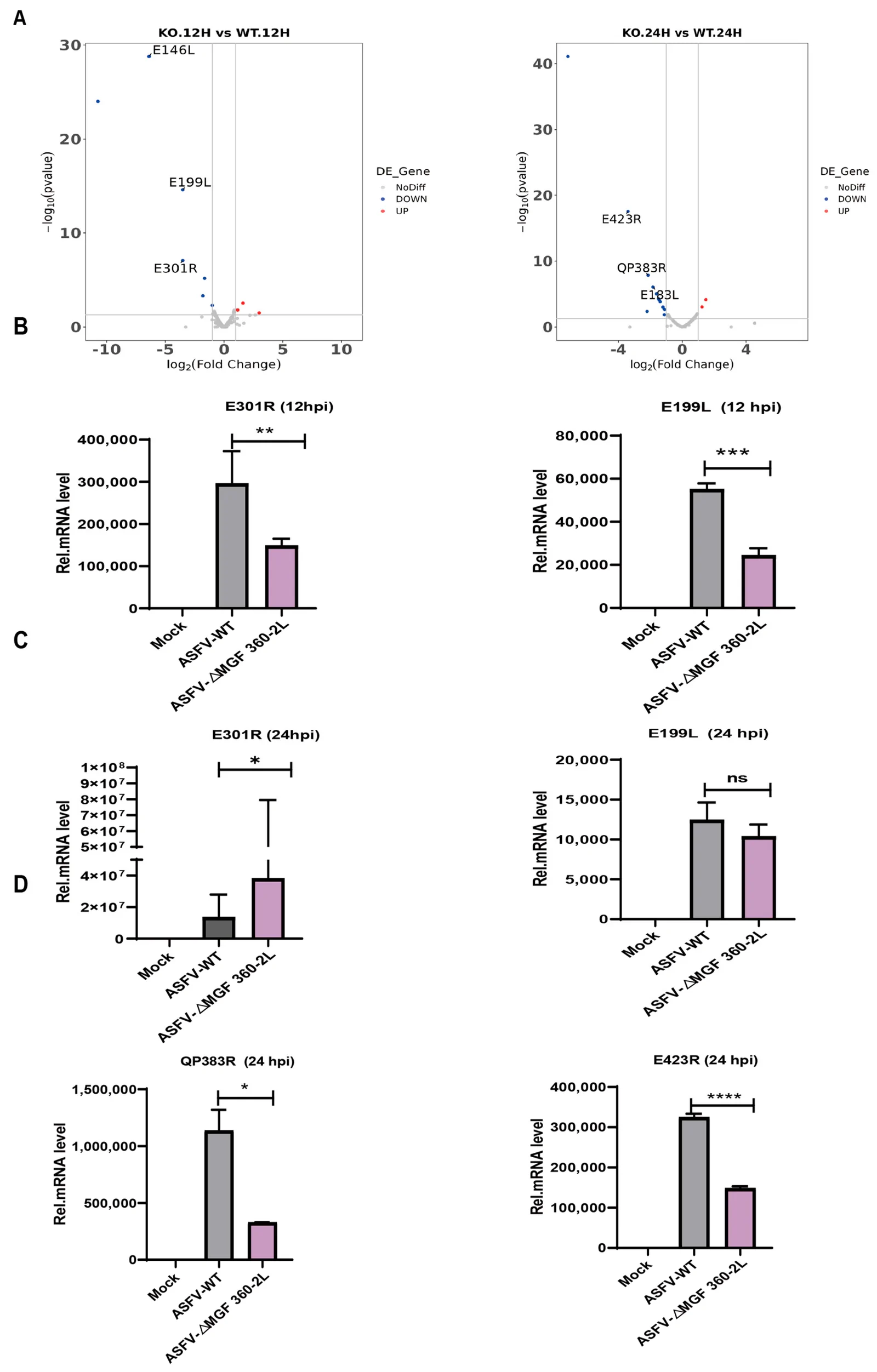
The DEGs of ASFV in PAMs from the ASFV CN/GS/2018-ΔMGF 360-2L infection group and the wild-type ASFV CN/GS/2018 infection group were analyzed and experimentally validated. (**A**) Differential ASFVgenes of ASFV CN/GS/2018-ΔMGF 360-2L infected or ASFV CN/GS/2018-infected PAMs were analyzed by RNA-seq. (**B**) The RT-qPCR technique was employed to validate and analyze the differential expression of the ASFV genes *E301R* and *E199L* in PAMs infected with either ASFV CN/GS/2018-ΔMGF 360-2L or the wild-type strain ASFV CN/GS/2018 for a duration of 12 h. (**C**) The RT-qPCR technique was employed to validate and analyze the differential expression of the ASFV genes *E301R* and *E199L* in PAMs infected with either ASFV CN/GS/2018-ΔMGF 360-2L or the wild-type strain ASFV CN/GS/2018 for a duration of 24 h. (**D**) The RT-qPCR technique was employed to validate and analyze the differential expression of the ASFV genes *QP383R* and *E423R* in PAMs infected with either ASFV CN/GS/2018-ΔMGF 360-2L or the wild-type strain ASFV CN/GS/2018 for a duration of 24 h. Data are presented as mean ± standard deviation (SD). Statistical differences between groups were assessed using Student’s *t*-test. * *p* < 0.05, ** *p* < 0.01, *** *p* < 0.001, **** *p* < 0.0001; no significant difference, *p* > 0.05.

To investigate whether the deletion of *MGF 360-2L* influences the expression of viral proteins, sequencing data from each sample were aligned and analyzed against the ASFV CN/GS/2018 reference sequence. The analysis revealed that at 12 hpi, the expression levels of 10 genes exhibited significant changes compared to the control group, including 3 up-regulated and 7 down-regulated genes. At 24 hpi, a total of 16 genes showed significant alterations in expression, with 2 gene up-regulated and the remaining 14 genes down-regulated (Log2FC > 1 or <−1 and padj < 0.05) (Figure 5A). Among the differentially expressed genes, ΔMGF 360-2L significantly downregulated the expression of *E301R* and *E199L*—two genes essential for the life cycle of ASFV—at 12 hpi (Figure 5B). Further observations indicated that by 24 hpi, the expression of *E301R* had increased, while the expression of *E199L* had partially recovered (Figure 5C). Such a regulatory strategy may allow the virus to maintain the host immune response within a finely tuned equilibrium—neither sufficiently robust to clear the infection nor excessively activated to induce severe inflammatory pathology. Meanwhile, at 24 h post-infection, *QP383R* and *E423R* exhibited significant down-regulation (Figure 5D).

## 4. Discussion

The ASFV genome encodes a large number of proteins, many of which remain functionally uncharacterized. Understanding these proteins is critical for elucidating viral replication mechanisms and pathogenicity, as well as for developing effective control strategies. In this study, we investigated the previously understudied *MGF 360-2L* gene and demonstrated that it is transcribed during the late phase of infection in PAMs (Figure 1E). The experiments conducted on the *MGF 360-2L* deletion mutant indicated that *MGF 360-2L* is not essential for the in vitro replication of the virus, as the deletion did not markedly compromise the replication capability of the ASFV GS/2018 strain in PAMs (Figure 1D). These findings suggest that the *MGF 360-2L* may possess redundant or context-dependent functions and could potentially interact with other viral genes to fulfill its role. This finding aligns with previous reports on the functional heterogeneity of ASFV MGF members, where deletions of certain *MGF* genes (e.g., some *MGF360* or *MGF505* members) attenuate viral virulence, while others exhibit no discernible impact on pathogenicity. Further studies are warranted to explore its role in natural infections or different host environments, particularly its potential involvement in immune evasion or host range determination.

Transcriptomic analysis of PAMs infected with ASFV-ΔMGF 360-2L or ASFV GS/2018 strain revealed significant enrichment of DEGs in innate immune pathways, particularly IFN-I signaling cascade. While previous studies have identified multiple ASFV-encoded proteins (e.g., *DP96R* [24], *E120R* [25], *MGF 505-7R* [26], and *pI215L* [27]) that suppress IFN-I production to evade host immunity. Our findings demonstrate that the presence of *MGF 360-2L* is associated with enhanced interferon-related transcriptional signatures. Compared to infection with the ASFV GS/2018 strain, the expression of multiple immune-related genes was markedly downregulated in PAMs following infection with the ASFV-ΔMGF 360-2L. The most significantly suppressed genes between 12 and 24 hpi included those encoding chemokines (e.g., *CXCL10* and *CXCL11*), type I interferons (*IFNA1* and *IFNB1*), and *SOCS1*, a key negative regulator of the JAK-STAT signaling pathway. Notably, despite these transcriptional changes, *MGF 360-2L* itself did not directly activate the IFN-I signaling pathway (Figure S3).

Furthermore, both RNA sequencing and quantitative PCR results indicated that at 12 hpi, the expression levels of two essential viral genes, *E199L* and *E301R*, were significantly reduced in the ΔMGF 360-2L infection group. By 24 hpi, *E301R* expression partially recovered, whereas *E199L* showed only limited restoration, suggesting that *MGF 360-2L* is critical for maintaining stable expression of these viral proteins.

In summary, this study reveals that *MGF 360-2L*, a late-expressed gene of ASFV, plays a pivotal role in modulating both host immune responses and viral gene expression. Its underlying mechanism appears to be complex and finely regulated. Future studies should aim to elucidate the specific molecular interactions through which *MGF 360-2L* influences the IFN-I signaling pathway and to clarify its functional roles and molecular networks during the viral replication cycle and pathogenesis.

## 5. Conclusions

In summary, this study elucidates the critical role of the ASFV late-expressed gene *MGF 360-2L* in the viral immune regulatory mechanism. Transcriptomic analysis reveals that the presence of *MGF 360-2L* is closely associated with the activation of interferon-related signaling pathways. Compared to the ASFV CN/GS/2018, the ASFV CN/GS/2018-ΔMGF360-2L leads to significant downregulation of multiple immune-related genes in PAMs, particularly between 12 and 24 hpi. These include key chemokines (*CXCL10*, *CXCL11*), type I interferons (*IFNA1*, *IFNB1*), and *SOCS1*, a negative regulator of the JAK-STAT signaling pathway. Furthermore, *MGF 360-2L* exhibits a strong correlation with the viral genes *E199L* and *E301R*, suggesting a potential role in the fine-tuning of viral protein expression and the execution of immune evasion strategies.

## Figures and Tables

**Table 1:** Primers were used to assay for insertion location and purity by PCR.

Primers	Sequence (5′–3′)	Description
**ΔMGF_360-2L** **-LA-F**	GAGTGCACCATATGTCGCGAATGCTCTCTTCGAAAGCATCAGCT	For ASFV CN/GS/2018
**ΔMGF_360-2L** **-LA-R**	TGGACGAGCTGTACAAGTAATTTACAGGCTTTATTAAAAAAAAA	
**ΔMGF_360-2L** **-RA-F**	GACTTTTCCTCCGGCGACCCGCTAGCATTTTTTTTTGCAAAATGGTTTAA	For ASFV CN/GS/2018
**ΔMGF_360-2L** **-RA-R**	ATAGGGAGACCGGCAGATCTTGATGGAGCTTTAGAAGCTGATAA	
**72EGFP-F**	TTACTTGTACAGCTCGTCCATGC	For ASFV CN/GS/2018
**72EGFP-R**	GGGTCGCCGGAGGAAAAGTCAAAA	
**L-ΔMGF_360-2L-LA-F**	AGAGAAGAGTCTGGACTGTAATT	Detect the insertion position of the left arm
**L-ΔMGF_360-2L-LA-R**	ATCATGGCCGACAAGCAGAAG	
**L-ΔMGF_360-2L-RA-F**	ATGGCGGTTTATGCGAAGGATCTT	Detect the insertion position of the right arm
**L-ΔMGF_360-2L-RA-R**	AGCGGTAAATTTATATAACAACC	
**P-ΔMGF_360-2L-F**	TCAAAGCTTTCCTCGCACCTAACT	Detect the purity
**P-ΔMGF_360-2L-R**	AGCTAATGCTTTCGAAGAGAGCTT	

F, forward; R, reverse.

**Table 2:** Primers were used to assay gene expression by real-time quantitative PCR.

Primers	Sequence (5′–3′)	Description
**B646L-F**	TCCGAACTTGTGCCAATCTC	For ASFV CN/GS/2018
**B646L-R**	CAACAATAACCACCACGATGA	
**CP204L-F**	CGGTCGTAACAATTCTACCGC	For ASFV CN/GS/2018
**CP204L-R**	CAAGTTGTGTTTCATGCGGG	
**MGF_360-2L**	AAATGATGTATCTGGCCTGTATGAG	For ASFV CN/GS/2018
**MGF_360-2L**	CCATTGCCCGATTGATGTTAG	
**GAPDH-F**	CCTTCATTGACCTCCACTACA	For ASFV CN/GS/2018
**GAPDH-R**	GATGGCCTTTCCATTGATGAC	

F, forward; R, reverse.

**Table 3:** Primers were used to validate differentially expressed transcripts by PCR.

Primers	Sequence (5′–3′)
**pID3-F**	GCATCTTCCCATCCAGACA
**pID3-R**	CAGTGGCAGAAGGTCGTT
**pSMAD9-F**	ATGGCTTTGAAGTCGTGTATGAG
**pSMAD9-R**	TGTGACATCCTGGCGGTGA
**pCOLEC12-F**	ACATCCGTTTGGATTCTGTTTC
**pCOLEC12-R**	GCTGTCCTCTGTCACCTTTCG
**pLpl-F**	CTGCCTGAAGTCTCCACAA
**pLpl-R**	ACATTAGCAACTCTCCAATATCCA
**pCXCL10-F**	TGCCCACATGTTGAGATCAT
**pCXCL10-R**	CGGCCCATCCTTATCAGTAG
**pCXCL11-F**	TTGCATTGGCCCTGGAGT
**pCXCL11-R**	TTGGGATTTAGGCATCTTCGT
**pINFA1-F**	CAGTTCTGCACTGGACTGGATC
**pINFA1-R**	CATGACTTCTGCCCTGACGAT
**pSDS-F**	CGTGAGAACCCCTATTCGTGA
**pSDS-R**	GCTGCCAAGCCTGCATTAC
**pSOCS1-F**	TGGCAGCCGACAATGCA
**pSOCS1-R**	GGAGGAGGATGAAGATGAAGAGG
**pE301R-F**	GTCGCAGGTGTTCCAGATAAA
**pE301R-R**	GGCATCCATTTCCGATTGAAAG
**pE199L-F**	GGGCAATATTTCCGACCTATAC
**pE199L-R**	GGGCAACTTATCGTCATTGT
**pE423R-F**	CTCCCGCAGGCAACAGAAA
**pE423R-R**	AGTGAACACGACATCCATCCC
**pQP383R-F**	ATCCGCCCGCTGATAGTTT
**pQP383R-R**	CCCAATCCTTGCATGACCTT

F, forward; R, reverse; p, porcine.

**Table 4:** Summary of raw sequencing data quality.

Sample	Raw Reads	Bases	GC (%)	Q20 (%)	Q30 (%)	Avg. Quality
**Mock**	42,538,766	6.381	52.695	98.595	95.345	39.04
**ASFV-WT-12hpi**	38,844,682	5.827	51.965	98.66	95.515	39.08
**ASFV-WT-24hpi**	46,905,862	7.036	51.79	98.28	94.92	38.93
**ASFV-ΔMGF-360-2L-12hpi**	45,805,112	6.871	52.365	98.475	95.205	39.005
**ASFV-ΔMGF-360-2L-24hpi**	46,288,930	6.943	51.605	98.435	95.215	39

**Table 5:** Statistics of data mapping results.

Sample	Total Reads After Filtered	Mapped on Reference	Unmapped	Multi-Mapped	Non-Splice Reads	Splice Reads
**MOCK**	38,912,094	36,827,092 (94.64%)	2,085,002 (5.36%)	1,935,985 (4.98%)	19,393,634 (49.84%)	15,497,473 (39.83%)
**ASFV-WT-12hpi**	35,852,066	32,824,312 (91.55%)	3,027,754 (8.45%)	1,482,865 (4.14%)	17,062,235 (47.59%)	14,279,212 (39.83%)
**ASFV-WT-24hpi**	42,290,538	36,766,781 (86.94%)	5,523,757 (13.06%)	2,025,063 (4.79%)	19,552,272 (46.23%)	15,189,446 (35.92%)
**ASFV-ΔMGF-360-2L-12hpi**	41,526,394	38,230,610 (92.06%)	3,295,784 (7.94%)	1,900,881 (4.58%)	19,633,253 (47.28%)	16,696,476 (40.21%)
**ASFV-ΔMGF-360-2L-24hpi**	42,190,368	37,017,952 (87.74%)	5,172,416 (12.26%)	2,070,076 (4.91%)	19,588,342 (46.43%)	15,359,534 (36.41%)

## Data Availability

The original contributions presented in this study are included in the article/Supplementary Material. Further inquiries can be directed to the corresponding author.

## Supplementary Materials

The following supporting information can be downloaded at: https://www.mdpi.com/article/10.3390/vaccines13090918/s1, Figure S1: (A) Schematic representation of the MGF 360-2L ORF region in the ASFV CN/GS/2018 genome, depicting adjacent open reading frames. (B) Prediction of transmembrane domains in the MGF 360-2L protein.; Figure S2: The sequencing results of the left and right homologous arms of ASFV CN/GS/2018-ΔMGF360-2L; Figure S3: Phosphorylated STAT1 in PAMs infected with ASFV CN/GS/2018-ΔMGF360-2L or ASFV CN/GS/2018 was examined by Western blotting analysis. PAMs infected with ASFV CN/GS/2018-ΔMGF360-2L or ASFV CN/GS/2018 for 12 hours, The cells were collected and lysed, and the phosphorylation of STAT1 was detected by Western blotting.

## Abbreviations

The following abbreviations are used in this manuscript:

ASF	African swine fever
ASFV	African swine fever virus
MGF	multi-gene family
DEGs	differentially expressed genes
GO	Gene Ontology
KEGG	Kyoto Encyclopedia of Genes and Genomes
IFN-I	Type I interferon
NCLDVs	nucleo-cytoplasmic large DNA viruses
dsDNA	enveloped double-stranded DNA
PAMs	Porcine alveolar macrophages
MOI	multiplicity of infection
QC	quality control
TCID_50_	the 50% effective infectious dose
BSL-3	biosafety level 3
LVRI	Lanzhou Veterinary Research Institute

## References

[1] Wang N., Zhao D., Wang J., Zhang Y., Wang M., Gao Y., Li F., Wang J., Bu Z., Rao Z. (2019). Architecture of African swine fever virus and implications for viral assembly. Science.

[2] Zhao D., Liu R., Zhang X., Li F., Wang J., Zhang J., Liu X., Wang L., Zhang J., Wu X. (2019). Replication and virulence in pigs of the first African swine fever virus isolated in China. Emerg. Microbes Infect..

[3] Pikalo J., Zani L., Hühr J., Beer M., Blome S. (2019). Pathogenesis of African swine fever in domestic pigs and European wild boar—Lessons learned from recent animal trials. Virus Res..

[4] Zhu Z., Mao R., Liu B., Liu H., Shi Z., Zhang K., Liu H., Zhang D., Liu J., Zhao Z. (2024). Single-cell profiling of African swine fever virus disease in the pig spleen reveals viral and host dynamics. Proc. Natl. Acad. Sci. USA.

[5] Gao X., Liu T., Liu Y., Xiao J., Wang H. (2021). Transmission of African swine fever in China Through Legal Trade of Live Pigs. Transbound. Emerg. Dis..

[6] Gaudreault N.N., Madden D.W., Wilson W.C., Trujillo J.D., Richt J.A. (2020). African Swine Fever Virus: An Emerging DNA Arbovirus. Front. Vet. Sci..

[7] Malogolovkin A., Kolbasov D. (2019). Genetic and antigenic diversity of African swine fever virus. Virus Res..

[8] Matsuyama T., Kiryu I., Mekata T., Takano T., Umeda K., Matsuura Y. (2023). Pathogenicity, genomic analysis and structure of abalone asfa-like virus: Evidence for classification in the family Asfarviridae. J. Gen. Virol..

[9] Zsak L., Sur J.H., Burrage T.G., Neilan J.G., Rock D.L. (2001). African Swine Fever Virus (Asfv) Multigene Families 360 and 530 Genes Promote Infected Macrophage Survival. Sci. World J..

[10] Zhu Z., Chen H., Liu L., Cao Y., Jiang T., Zou Y., Peng Y. (2021). Classification and characterization of multigene family proteins of African swine fever viruses. Brief. Bioinform..

[11] Portugal R., Coelho J., Höper D., Little N.S., Smithson C., Upton C., Martins C., Leitão A., Keil G.M. (2015). Related strains of African swine fever virus with different virulence: Genome comparison and analysis. J. Gen. Virol..

[12] Xiang Z., Xu Z., Weng W., Wang H., Wu J., Jiang F., Qu Y., Li Q., Gao P., Zhou L. (2025). The African swine fever virus MGF360-16R protein functions as a mitochondrial-dependent apoptosis inducer by competing with BAX to bind to the HSP60 protein. J. Virol..

[13] Chen Q., Wang X.X., Jiang S.W., Gao X.T., Huang S.Y., Liang Y., Jia H., Zhu H.F. (2023). MGF360-12L of ASFV-SY18 is an immune-evasion protein that inhibits host type I IFN, NF-κB, and JAK/STAT pathways. Pol. J. Vet. Sci..

[14] Wang Z., He Y., Huang Y., Zhai W., Tao C., Chu Y., Pang Z., Zhu H., Zhao P., Jia H. (2024). African swine fever virus MGF360-4L protein attenuates type I interferon response by suppressing the phosphorylation of IRF3. Front. Immunol..

[15] Zhang K., Yang B., Shen C., Zhang T., Hao Y., Zhang D., Liu H., Shi X., Li G., Yang J. (2022). MGF360-9L Is a Major Virulence Factor Associated with the African Swine Fever Virus by Antagonizing the JAK/STAT Signaling Pathway. mBio.

[16] Yang K., Xue Y., Niu H., Shi C., Cheng M., Wang J., Zou B., Wang J., Niu T., Bao M. (2022). African swine fever virus MGF360-11L negatively regulates cGAS-STING-mediated inhibition of type I interferon production. Vet. Res..

[17] Ilangumaran S., Rottapel R. (2003). Regulation of cytokine receptor signaling by SOCS1. Immunol. Rev..

[18] Matamoros T., Alejo A., Rodríguez J.M., Hernáez B., Guerra M., Fraile-Ramos A., Andrés G. (2020). African Swine Fever Virus Protein pE199L Mediates Virus Entry by Enabling Membrane Fusion and Core Penetration. mBio.

[19] Li S., Ge H., Li Y., Zhang K., Yu S., Cao H., Wang Y., Deng H., Li J., Dai J. (2023). The E301R protein of African swine fever virus functions as a sliding clamp involved in viral genome replication. mBio.

[20] García-Belmonte R., Pérez-Núñez D., Pittau M., Richt J.A., Revilla Y. (2019). African Swine Fever Virus Armenia/07 Virulent Strain Controls Interferon Beta Production through the cGAS-STING Pathway. J. Virol..

[21] Cao L., Li Q., Chen X. (2018). The HindIII and PvuII polymorphisms of lipoprotein lipase (LPL) gene reduce the risk of ischemic stroke (IS): A meta-analysis. Medicine.

[22] Massagué J., Wotton D. (2000). Transcriptional control by the TGF-beta/Smad signaling system. EMBO J..

[23] Ma Y.J., Hein E., Munthe-Fog L., Skjoedt M.O., Bayarri-Olmos R., Romani L., Garred P. (2015). Soluble Collectin-12 (CL-12) Is a Pattern Recognition Molecule Initiating Complement Activation via the Alternative Pathway. J. Immunol..

[24] Wang X., Wu J., Wu Y., Chen H., Zhang S., Li J., Xin T., Jia H., Hou S., Jiang Y. (2018). Inhibition of cGAS-STING-TBK1 signaling pathway by DP96R of ASFV China 2018/1. Biochem. Biophys. Res. Commun..

[25] Liu H., Zhu Z., Feng T., Ma Z., Xue Q., Wu P., Li P., Li S., Yang F., Cao W. (2021). African Swine Fever Virus E120R Protein Inhibits Interferon Beta Production by Interacting with IRF3 To Block Its Activation. J. Virol..

[26] Li J., Song J., Kang L., Huang L., Zhou S., Hu L., Zheng J., Li C., Zhang X., He X. (2021). pMGF505-7R determines pathogenicity of African swine fever virus infection by inhibiting IL-1β and type I IFN production. PLoS Pathog..

[27] Huang L., Xu W., Liu H., Xue M., Liu X., Zhang K., Hu L., Li J., Liu X., Xiang Z. (2021). African Swine Fever Virus pI215L Negatively Regulates cGAS-STING Signaling Pathway through Recruiting RNF138 to Inhibit K63-Linked Ubiquitination of TBK1. J. Immunol..

