# Correction: Zhang et al. African Swine Fever Virus MGF 360-2L Disrupts Host Antiviral Immunity Based on Transcriptomic Analysis. *Vaccines* 2025, *13*, 918

**DOI:** 10.3390/vaccines14020143

**Published:** 2026-01-30

**Authors:** Taoqing Zhang, Xiaodong Qin, Sujie Dong, Yuanshu Wu, Xiaolan Qi, Jingjing Ren, Yuan Wen, Zhengwang Shi, Tao Feng, Bingjie Sun, Changying Wang, Haixue Zheng

**Affiliations:** 1 State Key Laboratory for Animal Disease Control and Prevention, College of Veterinary Medicine, Lanzhou University, Lanzhou Veterinary Research Institute, Chinese Academy of Agricultural Sciences, Lanzhou 730046, China; 2 Gansu Province Research Center for Basic Disciplines of Pathogen Biology, Lanzhou 730046, China

In the original publication [1], there was a mistake in Figure 4, “Validation of RNA-Seq results by RT-qPCR”, as published. During the final stages of figure integration and layout, due to an oversight on our part, and because the expression trends of CXCL10 and CXCL11 were consistent (both downregulated) and aligned with the overall conclusions from the transcriptomic analysis, the two images were inadvertently set as the same one in Figure 4C. We have thoroughly re-examined all original data and confirmed that the data itself is accurate. This error was confined solely to the final presentation of the figures. The corrected Figure 4 appears below.

The authors state that the scientific conclusions are unaffected. This correction was approved by the Academic Editor. The original publication has also been updated.

## Figures and Tables

**Figure 4:**
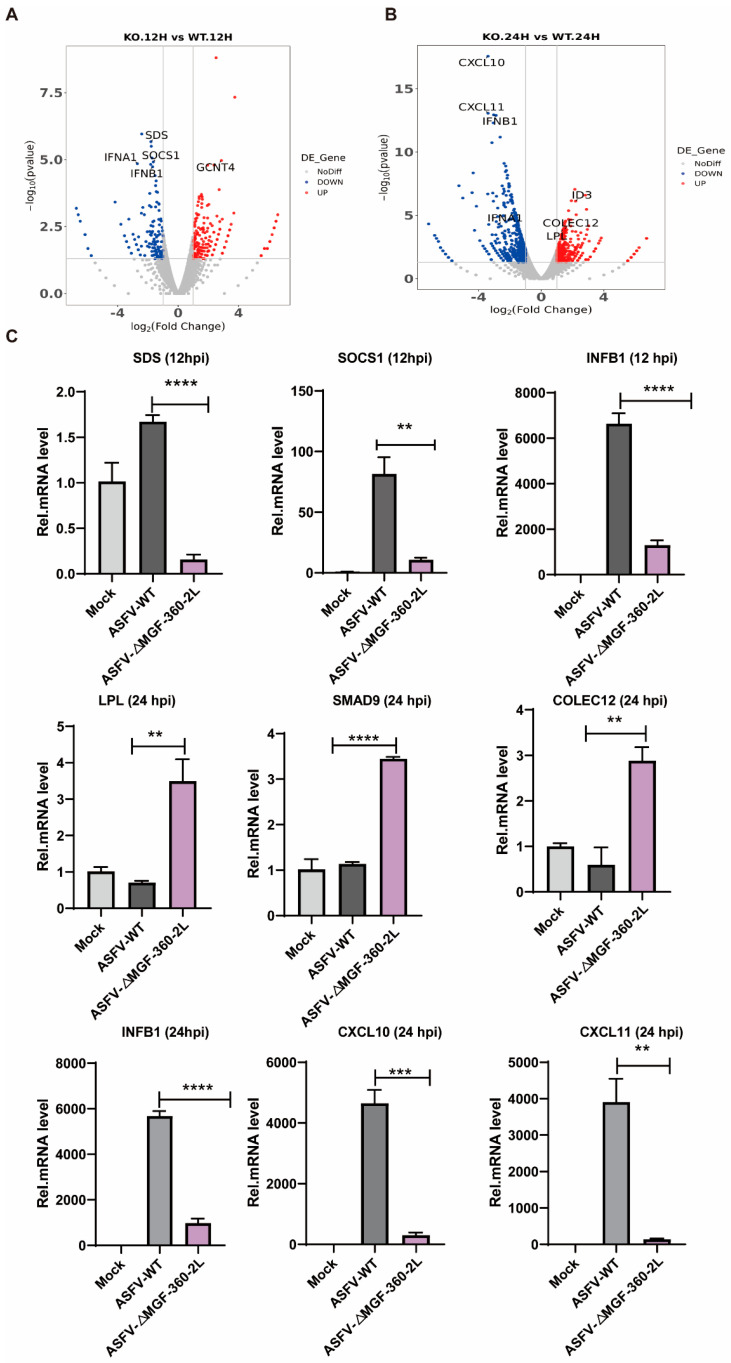
Validation of RNA-Seq results by RT-qPCR. (**A**) Volcano plot of ASFV CN/GS/2018-ΔMGF 360-2L vs. ASFV CN/GS/2018 at 12 hpi. (**B**) Volcano plot of ASFV CN/GS/2018-ΔMGF 360-2L vs. ASFV CN/GS/2018 at 24 hpi. (**C**) PAMs were uninfected or infected with ASFV CN/GS/2018 or ASFV CN/GS/2018-ΔMGF 360-2L at 1.0 MOI for 12 hpi or 24 hpi. RNA was extracted and RT-qPCR was carried to test their expression level. The data are presented as means with SD. *p*-values were analyzed by Student’s *t*-test. ** *p* <  0.01, *** *p* <  0.001, **** *p* < 0.0001 and non-significant at *p*  >  0.05.
