# Supplementary material for: African Swine Fever Virus MGF 360-2L Disrupts Host Antiviral Immunity Based on Transcriptomic Analysis
Source: Vaccines (Basel). 2025 Aug 28;13(9):918. doi: 10.3390/vaccines13090918 (PMC12474291; doi:10.3390/vaccines13090918)
Supplement: Supplementary file 1 [file vaccines-13-00918-s001.zip › vaccines-3806701-supplementary.pdf]

African swine fever virus MGF 360-2L disrupt host antiviral immunity based on transcriptomic analysis

A

B

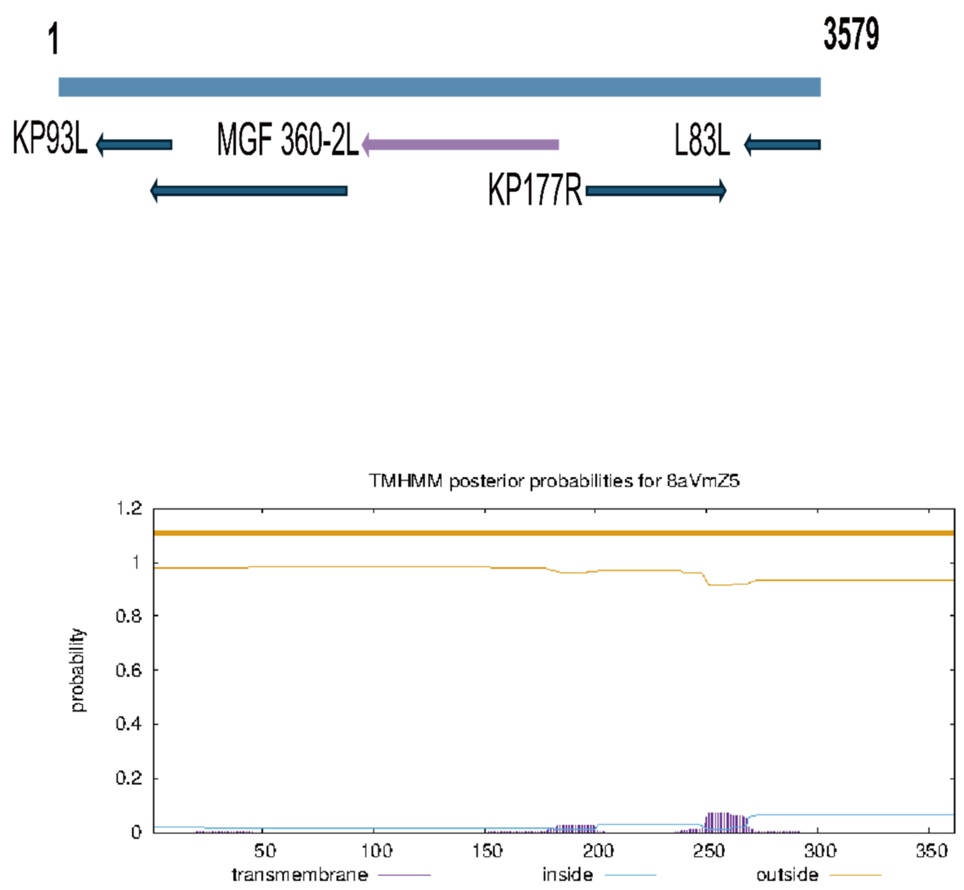

Figure S1. （A）Schematic representation of the MGF 360-2L ORF region in the ASFV CN/GS/2018 genome, depicting adjacent open reading frames. (B) Prediction of transmembrane domains in the MGF 360-2L protein.

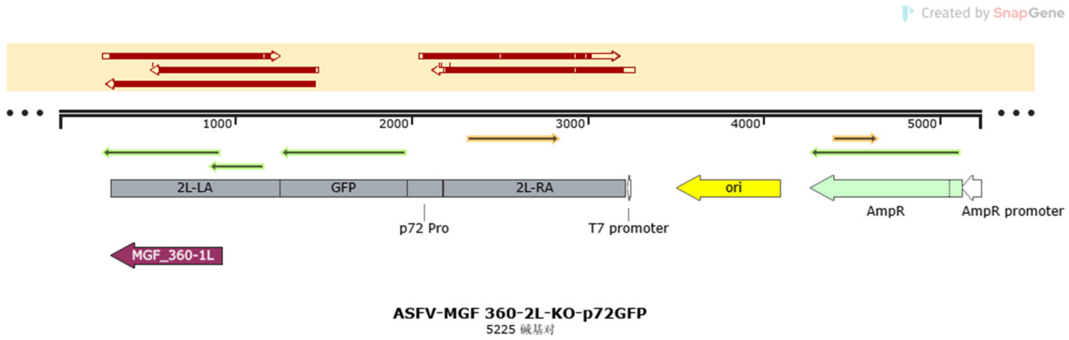

Figure S2. The sequencing results of the left and right homologous arms of ASFV CN/GS/2018-ΔMGF360-2L

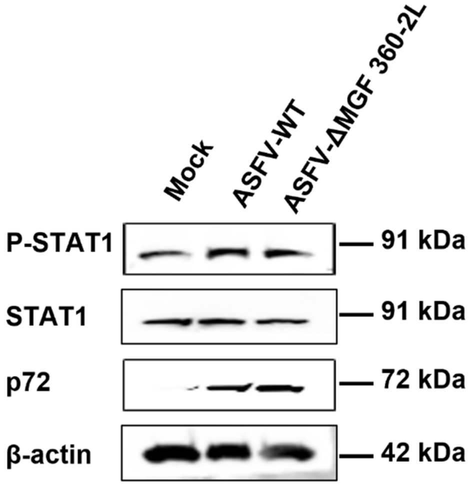

Figure S3. Phosphorylated STAT1 in PAMs infected with ASFV CN/GS/2018-ΔMGF360-2L or ASFV CN/GS/2018 was examined by Western blotting analysis. PAMs infected with ASFV CN/GS/2018-ΔMGF360-2L or ASFV CN/GS/2018 for 12 hours, The cells were collected and lysed, and the phosphorylation of STAT1 was detected by Western blotting .
